# Personalized Learning With Artificial Intelligence in Dental Education: An Integrative Review

**DOI:** 10.1155/ijod/8928166

**Published:** 2026-01-16

**Authors:** Mithun Pai BH, Shweta Yellapurkar, Kavery Chengappa, Kalyana C. Pentapati

**Affiliations:** ^1^ Department of Public Health Dentistry, Manipal College of Dental Sciences Mangalore, Manipal Academy of Higher Education, Manipal, 576104, Karnataka, India, manipal.edu; ^2^ Department of Oral Pathology and Microbiology, Manipal College of Dental Sciences Mangalore, Manipal Academy of Higher Education, Manipal, 576104, Karnataka, India, manipal.edu; ^3^ Department of Public Health Dentistry, Manipal College of Dental Sciences, Manipal Academy of Higher Education, Manipal, India, manipal.edu

## Abstract

**Background:**

Dental education is at the pinnacle of innovation and evolution. Advancements in artificial intelligence (AI) have reinforced personalized dental education to learners’ preferences and needs to emerge as a game changer. Conventional dental education has rigid and standardized methods of learning that are inconvenient to diverse learning preferences. With the aid of AI, the learning process can be personalized. This integrative review discusses the various AI tool driving change in dental education to provide a tailor‐made learning experience for developing new knowledge that can inform practice and policy.

**Methods:**

The search strategy involved looking for keywords, “personalized learning” OR “adaptive learning” AND “artificial intelligence” AND “dental education” across databases. The results obtained were subject to review and the various AI tools used in dental education for personalizing learning process are discussed.

**Results:**

The keyword search across databases yielded 920 studies. After the 355 duplicates were removed 565 studies remained. The title and abstract screening were conducted; 377 studies were excluded resulting in 188 studies, 175 studies not complying with the inclusion criteria, resulting in 13 studies of which six were included as other studies did not specify on dental education, virtual simulation, augmented reality (AR) without inculcating AI technologies. The studies reviewed showed numerous tools such as virtual reality (VR) and AR being utilized for interactive and immersive engagement of learners.

**Conclusion:**

This review highlights both the pros and cons of AI‐driven personalized dental education and analyses prospects for improvement such as multistakeholder collaboration to create adaptive and student‐centered learning experience. Personalized dental education driven by AI can serve as a game changer in dental education by integrating technology for enhanced learning and development of policies for proper adaptation of such systems in personalized dental education.

## 1. Introduction

Learning of any kind varies by subjective aptitudes and individual predilections. For holistic learning, education that is tailored to one’s level of understanding and actively engages the learner, fosters great results. From integrating clinical and academic knowledge, incorporating advanced digital tools, to including artificial intelligence (AI), dental education has evolved substantially over the last few decades [1]. For a long time, the focus of dental education was on rigid, curriculum‐based, standardized, and one‐size‐fits‐all approach. With the wider availability and acceptance of dental research, there has been an inevitable change in dental education modelrom treatment‐centered to evidence‐based, clinical application‐based learning, involving cutting‐edge advances [2]. Paramount to the entire process of acquiring dental education has been the reliance on evidence‐based clinical practice, as the guiding pillar of the learning process [3].

Dental education has traditionally involved theoretical knowledge of basic medical science, dental sciences, along with the requisite clinical skills. The core competencies have continued to prioritize dental care delivery based on patient needs and an in‐depth understanding of oral health care needs of individuals and communities [4]. One of the key elements of dental training involves carrying out clinical procedures on patients under academic supervision. Preclinical training is provided to acquaint the students with the clinical procedures [5]. The dental curriculum has grown profoundly concordant with the innovations of contemporary times such as CAD/CAM, AI‐powered applications, intraoral scanners and such others [6].

Dental students like most other young learners belong to varied backgrounds, with differing cognitive abilities and experiences. The patient populations are constituted of individuals from diverse cultural backgrounds and require dental graduates to set aside their prejudices and embody cultural competence in dealing with such a varied population [7]. Owing to all these challenges in the learning process, dental students undergo immense stress and mental health issues [8].

The standardized methods employed in imparting dental education, results in students being disconnected with the learning process, specific student needs being unmet, perpetuating disparities in dental education. Clearly, there is a need for dental education that is adaptive to individual student needs while adhering to systematic knowledge and skill dissemination [9].

The United States Department of Education defines personalization of learning as, “instruction that is paced to learning needs, tailored to learning preferences, and tailored to the specific interests of different learners.” This all‐encompassing definition clearly indicates that the learner is at the center of the entire learning process [10]. Personalized learning conforms to individualistic interests, aptitude, modifying the instruction with respect to individual learner’s caliber [11].

Personalized learning has been in practice for a long time in different forms. In the early 18^th^ century, it was extensively practiced as private tutoring and apprenticeship, which was exclusive for the upper classes [12]. In later times, philosopher John Dewey [13] proposed the educational philosophy wherein education was centered around the capabilities and interests of the learner, based on hands‐on learning adapted to individualized needs.

As education evolved for the masses, the economic burden of meeting this demand led to the establishment of schools. John Lancaster, an educational reformer devised the “monitorial system,” wherein the proficient students taught the less proficient [12]. Further, Benjamin Bloom’s mastery learning model also promoted personalized learning by advancing individual students only after they achieved a level of mastery [14]. With the rapid technological advancements, personalized learning has shifted to digital aided systems. It has been integrated into various higher education models and teaching systems, including dentistry.

Conventional educational models have not been entirely adaptable and versatile as needed for personalized learning. This shortfall necessitates the use of newer mediums to personalize the learning experiences. Starting with digitalization of dental education and incorporation of technology in learning process, newer advancements have aided in personalizing the learning experience. One of the promising tools that can aid in this aspect is the use ofAI. AI has been adopted to various fields and at differing levels of education, it not only simplifies challenges but also opens newer paradigms [15]. One of these prospects is adapting or personalizing the education process to individual’s needs. Studies have shown the utility of AI in personalizing learning through conversational agents, personalized assessment and feedback systems, smart classrooms, virtual reality (VR), and learning analytics [16]. Moreover, the use of AI was shown to bring about a positive impact on student performances by improving learning outcomes, motivation and students attitudes towards learning. Application of AI tools has been shown to create tailored learning environment for students while enabling teachers to become facilitators of the learning process [17]. There is ample evidence on the significant role of AI in personalized learning, however there is a paucity of literature on the scope of AI in personalizing dental education. Thus, “this integrated review aims to critically examine the potential of personalized learning through AI in dental education by exploring its theoretical foundations, practical applications, and associated benefits and challenges which will further pave way for implication of policies on the integration of AI in personalized dental education.”

## 2. Methods

### 2.1. Search Strategy and Selection Criteria

The search strategy involved the use of following keywords along with Boolean operators: (“personalized learning” OR “adaptive learning”) AND (“artificial intelligence”) AND (“dental education”). The databases PubMed, Google Scholar, Scopus and Web of Science were searched. Studies were included from 2015 onwards. The studies included were reviews, original research, systematic reviews, and qualitative research. Only full‐text articles available in English language were included. Studies published in other languages, lacking full text and irrelevant to the scope of this review were excluded. The screening process was conducted by pairs of authors (Kavery Chengappa and Mithun Pai) who independently reviewed titles, abstracts, and full texts. Discrepancies were resolved by consensus or with a third author’s input (Shweta Yellapurkar). The start of search date of 2015 was selected to focus on the rising number of publications on this topic over the last 10 years. The search strategy was formed in conjunction with an experienced researcher (Kalyana C Pentapati) to ensure all relevant records in these databases were identified. Manual searching of the references for related studies was also carried out by searching in relevant high impact journals and citation trailing of relevant articles. Only studies that directly discussed the application of AI in personalizing dental education were included.

## 3. Results

**Figure 1:**
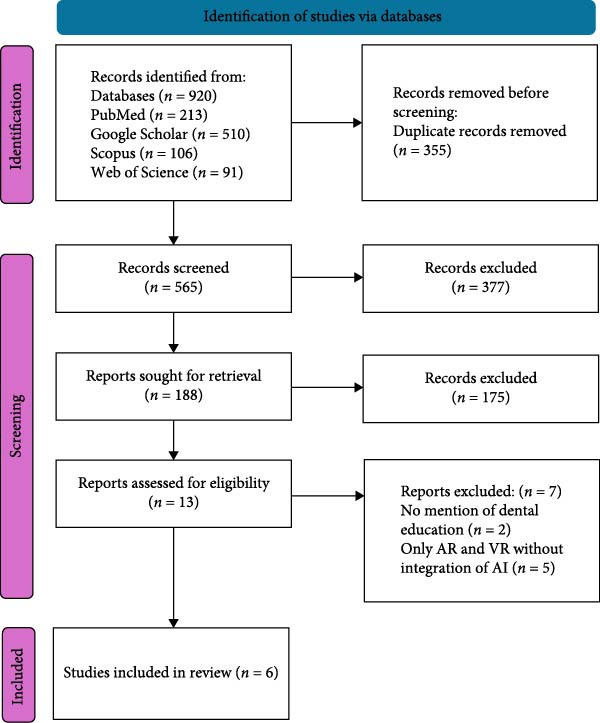
The PRISMA 2020 flowchart for the literature search process.

The keyword search across databases yielded 920 studies. After the 355 duplicates were removed 565 studies remained. The title and abstract screening were conducted; 377 studies were excluded resulting in 188 studies. 175 Studies not complying with the inclusion criteria, published in languages other than English, lacking full text were eliminated, resulting in 13 studies that were included for data synthesis. Further during data synthesis process, the studies that either did not specify on dental education or those that discussed virtual simulation, augmented reality (AR) without inculcating AI technologies were excluded from the review. Thus, finally yielding a total of six studies (Figure 1).

Of the six studies included, four studies focused on application of large language models (LLM) to personalizing dental education, while one study discussed incorporating machine learning (ML) model and another study briefly highlighted all AI technologies utilized in this aspect. Three of the studies followed cross‐sectional study design, two were mixed methods study and one study included was a narrative review (Table 1).

**Table 1:** A summary table of the finalized six studies.

Sl no.	Author and year	Reference number	Study design	AI technology in focus	Findings	Implication on personalized learning	Limitations identified by the study
1.	Danesh et al. (2023)	[18]	Analytical cross‐sectional	Artificial intelligence language models (ChatGPT3.5 and ChatGPT4) were assessed in a board‐style dental knowledge assessment	ChatGPT3.5 answered 61.3% questions correctly, while ChatGPT4 answered 76.9% correctly	ChatGPT4 produced accurate results and can be a complementary tool in dental education	• Prompts used were only text based and no image‐based questions. • ChatGPT4 is not a completely free version • Provided longer response in case of incorrect answers—posing risk of misinformation. • Real‐life scenario can differ.

2.	Sabri et al. (2024)	[19]	Comparative cross‐ sectional study	Performance of large language models (ChatGPT3.5, ChatGPT4 and Google Gemini) were compared with periodontal residents in terms of American academy of periodontology (AAP) in‐service exam questions from 2020 to 2023.	ChatGPT4 had the highest performance (79.57%) followed by Google Gemini (72.86%) and third year periodontal residents (69.06% ± 30.45)	Their accuracy implied these tools can be used for personalized tutoring to emphasize on individualistic interests and weak areas	• No image‐based questions included. • Inconsistent results for questions across years seen in case of ChatGPT3.5.

3.	Kavadella et al. (2024)	[20]	Mixed‐ methods study	ChatGPT’s application in dental education assessed in learning assignment by students and their experience evaluated qualitatively	Students who integrated ChatGPT in the learning process showed significantly better performance. In responses for evaluation questionnaire the students highlighted the benefits, limitations and prospects of ChatGPT	Personalized feedback and support provided by ChatGPT in the learning process.	The responses were found to be general, inaccurate with reliance on the phrasing of the prompt.

4.	Shoaib et al. (2024)	[21]	Descriptive cross‐sectional study	Decision tree machine learning model was used to map dental students preferred learning styles with instructional strategies	The decision tree model was able to provide appropriate instructional strategies for individual dental students. The model also showed good sensitivity, specificity along with precision	Helps in planning personalized student‐centered learning experience	Reduced generalizability, risk of overfitting by the model, and data‐privacy risks.

5.	Dutta et al. (2024)	[22]	Narrative review	AI in dental education, patient care and diagnostic precision	Simulation based learning systems, intelligent tutoring system, assistance with academic manuscripts and literature reviews can improve academic performance	The AI tools mentioned facilitate improvement in student performance by individualizing learning experience	Lack of data privacy, inherent biases within AI systems, and lack of regulations governing their use.

6.	Rath (2025)	[23]	Mixed ‐methods study	Application of Google Gemini in personalizing 3^rd^ year dental students learning assessed through quantitative and qualitative methods	15% increase in the scores of the students was observed after integrating Google Gemini Positive co relation between use of Google Gemini and performance of students Technical challenge in initial use, active student engagement, personalized feedback and suggest to expand the usage were the themes identified from the interview	Beneficial for formative learning in dental students	Summative assessment did not exhibit significant improvement. Long‐term effects and application in larger groups could not be established.

Critical appraisal of the studies adopted the suitable checklist as per the study design of the included studies. For cross‐sectional studies the Joanna Briggs Institute Critical Appraisal Checklist for Cross Sectional Studies [24] was used, for mixed methods study, the Mixed Methods Appraisal Tool (MMAT), Version 2018 [25] was applied and for narrative review, the Scale for the Assessment of Narrative Review Articles (SANRA) [26] was applied. On critical appraisal of the studies, the narrative review by Dutta et al. [22] obtained a low score of three out of a total of 12 for the six criteria assessed. All the cross‐sectional studies [18, 19, 21] showed high quality, with all criteria being met, excluding only the criteria of identifying and addressing confounding factors. Both the mixed methods study [20, 23] also showed consistently high quality with a lack only in accounting for confounders.

## 4. Discussion

AI has driven changes in dental education and continues to do so with newer applications and tools evolving at a rapid pace. Numerous AI tools have been utilized to personalize learning across subject areas. These include adaptive learning environments, personalized assessment and feedback systems, LLMs, intelligent tutorial and conversational systems, VR and AR for interactive and immersive engagement of learners [16]. The various application of AI in dental education and its role in personalizing the learning process has been discussed along with the user experiences and limitations encountered in these studies. The broader context and potential application of AI technologies have also been discussed.

### 4.1. LLMs for Personalized Dental Education

LLMs are AI models that are pre‐trained with large datasets from the internet, to generate coherent responses when given prompts. One of the popular LLM’s extensively used today, ChatGPT (chat generative pre‐trained transformer) developed by OpenAI, has a conversational interface. Other popular multimodal LLM’s include Google’s Gemini, Llama by Meta, DeepSeek. With its comprehensive problem‐solving abilities, LLM’s have emerged as a sought‐after aid by students and academicians alike [27]. Their potential applications span across different areas of learning making them easy to adopt in personalizing dental education. Interactive tutoring with real‐time feedback mechanisms, can be personalized to the learner’s needs and pace for better subject understanding [20]. Studies have shown that certain LLM’s exhibited an accuracy ranging from 78.80% to 80.98% in answering dental examinations, indicating their potential in being used as an educational tool that can aid in knowledge reinforcement [19]. The vast knowledge base and extensive responses by LLM’s such as ChatGPT can be effectively employed in simplifying complex concepts to address the learner’s queries [18]. Various studies assessed in this review evaluated the performance of different versions of ChatGPT, compared their efficiency with conventional study methods and have also evaluated other LLMs such as Google’s Gemini [18–20, 23].

While ChatGPT4 (76.9%) performed better than ChatGPT3.5 (61.3%) in a board‐style dental knowledge assessment [18]. The high performance of ChatGPT4 implies their use as a supplementary means to improvise student performance in dental board exams. However, the lack of image‐based questions in assessment and the absence of real‐life implementation scenario raises doubts on their accuracy. In another study, ChatGPT4 also showed highest performance (79.57%) followed by Google Gemini (72.86%) in comparison to periodontal residents (69.06% ± 30.45) in American Academy of Periodontology (AAP) in‐service exam questions from 2020 to 2023 [19]. This study also showed a lack of image‐based questions for assessment. In a mixed methods study, dental students integrated ChatGPT in the learning process showed significantly improved performance than those who used conventional methods [20]. The high reliance on phrasing of the prompt observed in this study, indicates the need for caution in use of AI tools. Integrating Google Gemini in 3^rd^ year dental students learning process not only helped in personalizing the process but also showed a 15% increase in the scores of these students [23].

### 4.2. VR and AR for Personalized Dental Training

In dental education, simulation of clinical scenarios is critical for developing skills among students in order to be proficient prior to treating actual patients. Contrary to conventional dental education that employed mannequin heads for simulation, VR offers practical motor skill development by providing tooth preparation simulations and surgical skill development [28]. Studies have shown that various VR/AR devices that are utilized to improve student performances have shown promising results. DentSim, which has both AR and VR to inculcate ergonomic postures, provides instant feedback and exam simulations [29]. Simodont VR simulator enables to practice dental procedures using the haptic technology that replicates the tactile perception of real teeth. It notably tailors the training process by including patient‐specific cases, 3D visualization and offering automated feedback [30]. VR simulation has been shown to improve clinical skills such as tooth preparation as compared to traditional training, consequentially increasing grades [31].

AR, wherein a virtual scenario is superimposed over a real‐world scenario resulting in a new perception, enables interactive learning. It can be utilized for developing three‐dimensional guidance of clinical procedures in real‐time [28]. The interactive set‐up, objective evaluation and uninterrupted access provided by VR and AR systems enables learners to personalize the learning systems to their needs. Further, integration of AI into these immersive technologies can further enhance the personalization of dental education through customized feedbacks. However, studies have demonstrated their effectiveness in preclinical courses such as tooth preparation, surgical practice, and anesthetic administration. But their efficiency in terms of learning dental implantology and dental anatomy has been inconclusive [32].

### 4.3. Personalized Assessment and Feedback Mechanisms

Intelligent tutoring systems are AI tools that provide educational material along with personalized feedback and guidance on a one‐on‐one basis akin to a human tutor [33]. These systems are enabled to determine students’ level of motivation, psychological state and cognitive form and its impact on their performance, offering subjective feedback, which helps identify flaws, and provides scope for improvement. When integrated with VR/AR systems, intelligent tutoring systems can facilitate better learning outcomes, greater academic and clinical performance [34].

### 4.4. ML

ML, a field of AI, comprises of machines learning datasets and performing tasks without being instructed humanly. In dental education, ML plays a critical role in the functioning of adaptive learning platforms [35]. ML systems can predict outcomes and generate hypotheses using the learned algorithms. This very function comes to the fore in assessing student performance and recommending case‐based scenarios, interactive exercises customized to the learner’s competence [22]. A recent study demonstrated that a decision tree ML tool had the ability to rapidly align students’ learning styles to the most suitable instructional strategies. Thus, personalizing learning content to individual preferences of each student [21]. The study revealed that the model was able to map suitable, individualized instructional strategies based on the learning styles of the students. The 100% accuracy and high sensitivity, specificity showed that the results were reliable.

### 4.5. Student’s Perceptions on use of AI Tools

Two studies assessed students’ perceptions following their use of AI tools wherein the students indicated initial challenge in technicalities of use, active engagement, personalized feedback, and suggested expansion of its use [23]. The students assessed in another study found there were discrepancy in certain result obtained by the usage of LLMs, inconsistent results and lack of criticality in the answers. The students suggested they could serve as aids in improving their performance but only to be used with critical interpretation [20].

### 4.6. Ethical Concerns and Practical Challenges

The potential of AI in personalizing dental education to meet the learners demands and preferences are largely promising. With research in this direction progressing by the day, consideration of the practical and ethical issues regarding its adoption must be simultaneously scrutinized.

One of the major drawbacks of an emerging technology such as AI is the usage of student datasets employed in training the systems. Datasets on student performance, behaviors and biometric data in simulator tools pose the risk of privacy and data safety. Compliance with regional and international data protection laws such as the General Data Protection Regulation (GDPR) and the Family Educational Rights and Privacy Act (FERPA) which uphold data protection and ensure data privacy must be mandated [36, 37].

The large datasets used for training AI systems may be subject to inherent bias in the algorithm. Inadequate representation of marginalized or minority student datasets in the algorithm may harbor inequality by homogenizing outcomes. Thus, violating the very foundation of personalized dental education [38].

With AI data sourced from databases, concerns of data ownership, usage permissions and modification rights present a challenge to intellectual property rights. This requires vigilant monitoring by dental institutions before implementing AI tools for learning [36]. The implementation of AI for personalizing dental education should incorporate methods such as data anonymization of student‐related personal data, are essential to ensure data safety. Further algorithmic transparency to clarify the underlying working of AI tools can enable to understand inherent flaws and biases of AI systems.

### 4.7. Addressing the Challenges

Ethical and practical challenges of integrating AI in dental education are well founded, however, that does not invalidate the enormous possibilities introduced by AI in personalizing dental education [37, 38]. This necessitates constant modifications and frequent revisions with human supervision to maintain ethical and academic integrity. Including datasets that are diverse in training data and repeated oversight of the algorithmic use can ensure the elimination of inherent bias in AI tools. Dental school educators should be trained and proficient in monitoring student usage of AI to prevent overuse and eliminate over‐reliance on AI. Accountability systems should be put in place by dental institutions and state administrations to ensure accountability and transparency in use of large student data.

### 4.8. Limitations

The studies included had smaller sample size and lacked generalizability. This integrative review presents with limitations. The studies could not be compared systematically due to lack of uniform assessment criteria for personalization in dental education driven by AI. The evolution of AI based tools being studied and developed constantly implies that the advancements discussed in this review could become obsolete in a short time, hampering the long‐term relevance of this review.

### 4.9. Way Forward

Further research is important to explore AI tools in dental education, with larger sample sizes, pilot studies, and long‐term evaluations of student performance. Faculty development programs are necessary to equip educators with the skills to integrate AI effectively into the curriculum. This integration must align to deliver high‐quality dental education while catering to diverse learning needs. A collaborative approach involving policymakers, educators, and technologists is crucial to overcome current implementation challenges. Ultimately, creating student‐centered, transparent AI‐driven learning environments with appropriate technological infrastructure is vital for advancing dental education.

## 5. Conclusion

This review emphasizes the promise of improving student engagement and customizing learning using LLMs such as ChatGPT and Google Gemini in the context of dental education. Looking at the generalizability or the accuracy of the provided information is important, but it is equally important to AI’s role in motivating self‐directed learners. The adaptiveness of dental education based on learners’ interests and proficiencies can enhance effectiveness and equip learners to tackle complex real‐world problems. In summary, with the appropriate technological applications, there is a revolutionary opportunity to reshape AI‐driven personalized education in dental training, integrating technology and healthcare for enhanced learning, and development of policies for proper adaptation of such systems in dental education.

## Ethics Statement

The authors have nothing to report.

## Consent

The authors have nothing to report.

## Conflicts of Interest

The authors declare no conflicts of interest.

## Author Contributions

**Mithun Pai BH, Shweta Yellapurkar**, **Kavery Chengappa, and Kalyana C. Pentapati:** conceptualization and design, acquisition, analysis, and interpretation of data, drafting and critically revising.

## Funding

This study is self‐funded and no external funding is received.

## Supporting Information

Additional supporting information can be found online in the Supporting Information section.

## Supporting information


**Supporting Information** Table contains the outcomes of each article and is critically appraised and described Jbi Critical Appraisal Checklist For Cross Sectional Studies. Mixed Methods Appraisal Tool (MMAT), version 2018. SANRA Checklist for Critical Appraisal of Narrative Reviews applied to the narrative review by Dutta et al. [22].

## Data Availability

No datasets were generated or analyzed during the current study.
