# Supplementary material for: Personalized Learning With Artificial Intelligence in Dental Education: An Integrative Review
Source: Int J Dent. 2026 Jan 16;2026:8928166. doi: 10.1155/ijod/8928166 (PMC12810715; doi:10.1155/ijod/8928166)
Supplement: Supplementary file 1 — Supporting Information Table contains the outcomes of each article and is critically appraised and described Jbi Critical Appraisal Checklist For Cross Sectional Studies. Mixed Methods Appraisal Tool (MMAT), version 2018. SANRA Checklist for Critical Appraisal of Narrative Reviews applied to the narrative review by Dutta et al. [22]. [file IJOD-2026-8928166-s001.docx]

**JBI CRITICAL APPRAISAL CHECKLIST FOR CROSS SECTIONAL STUDIES**

| Sl no. | Criteria |  | Study 1  Danesh A et al, 2023 | | Study 2  Sabri H et al, 2024 | Study 3  Shoaib A et al, 2024 |
| --- | --- | --- | --- | --- | --- | --- |
| 1 | Were the criteria for inclusion in the sample clearly defined? | Yes | 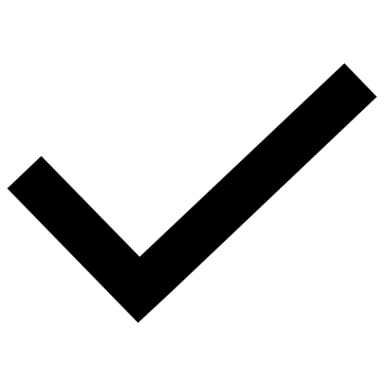 | | 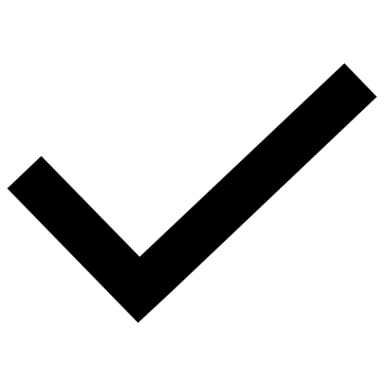 | 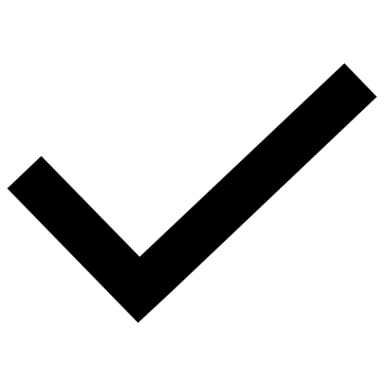 |
|  |  | No |  | |  |  |
|  |  | Unclear |  | |  |  |
|  |  | Not applicable |  | |  |  |
| 2. | Were the study subjects and the setting described in detail? | Yes |  | | 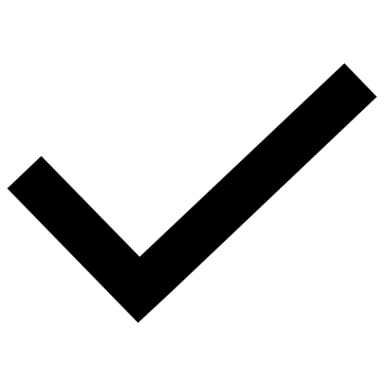 | 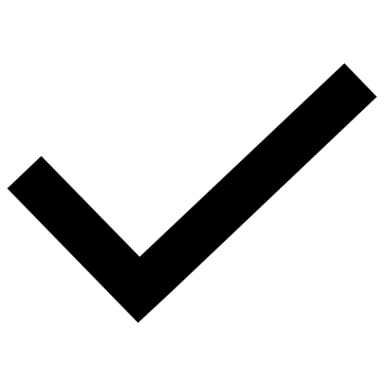 |
|  |  | No |  | |  |  |
|  |  | Unclear |  | |  |  |
|  |  | Not applicable | 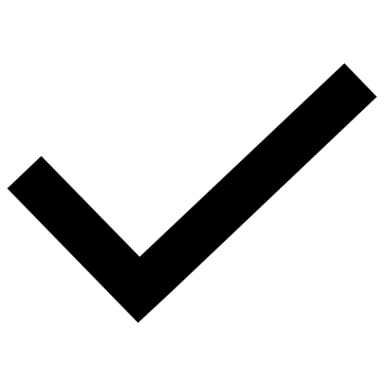 | |  |  |
| 3. | Was the exposure measured in a valid and reliable way? | Yes | 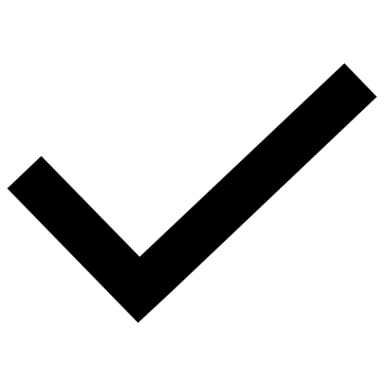 | | 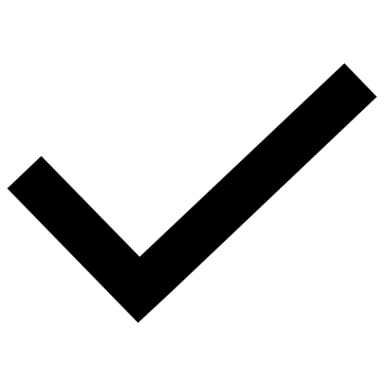 | 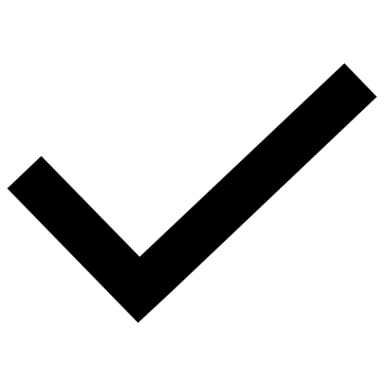 |
|  |  | No |  | |  |  |
|  |  | Unclear |  | |  |  |
|  |  | Not applicable |  | |  |  |
| 4. | Were objective, standard criteria used for measurement of the condition? | Yes | 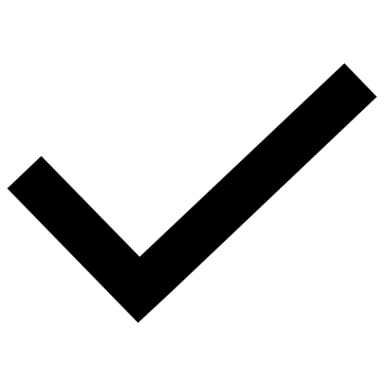 | | 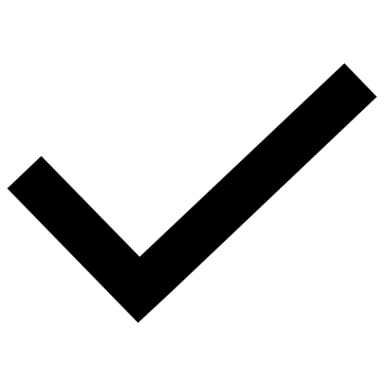 | 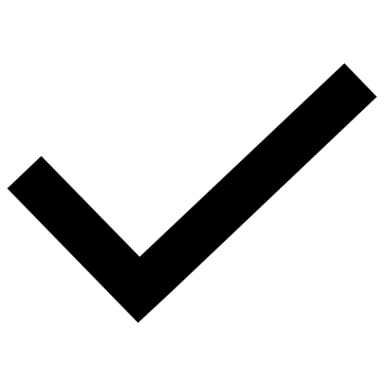 |
|  |  | No |  | |  |  |
|  |  | Unclear |  | |  |  |
|  |  | Not applicable |  | |  |  |
| 5. | Were confounding factors identified? | Yes |  | |  |  |
|  |  | No | 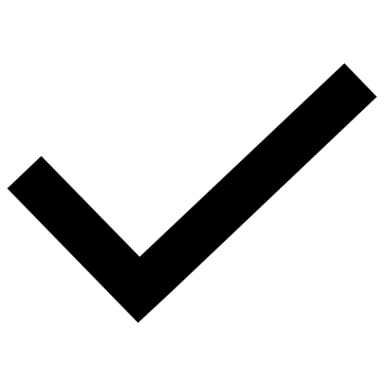 | | 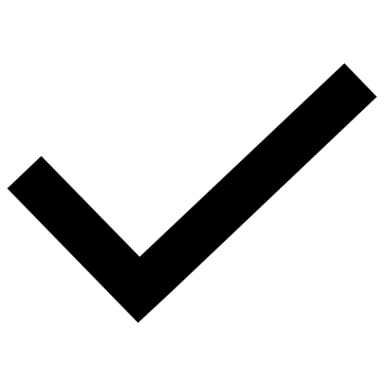 | 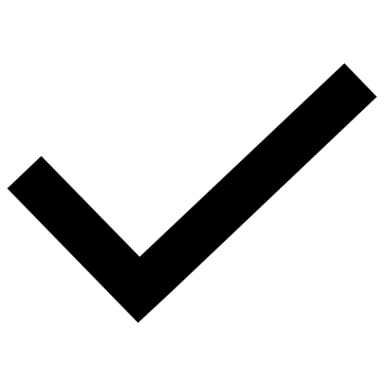 |
|  |  | Unclear |  | |  |  |
|  |  | Not applicable |  | |  |  |
| 6. | Were strategies to deal with confounding factors stated? | Yes | |  |  |  |
|  |  | No | | 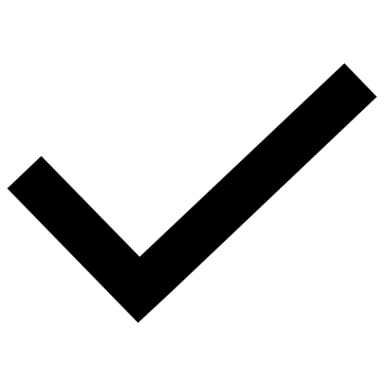 | 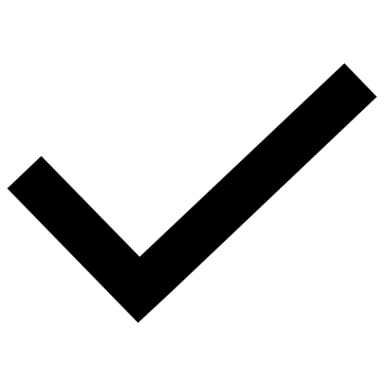 | 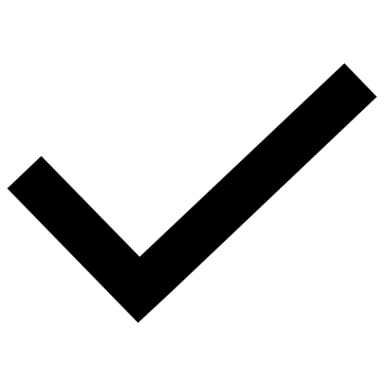 |
|  |  | Unclear | |  |  |  |
|  |  | Not applicable | |  |  |  |
| 7. | Were the outcomes measured in a valid and reliable way? | Yes | | 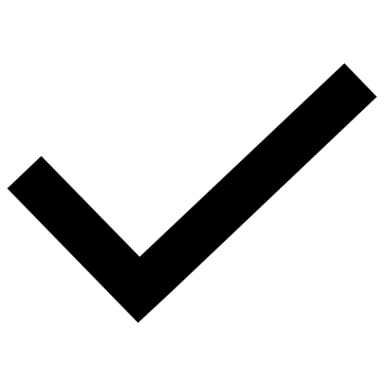 | 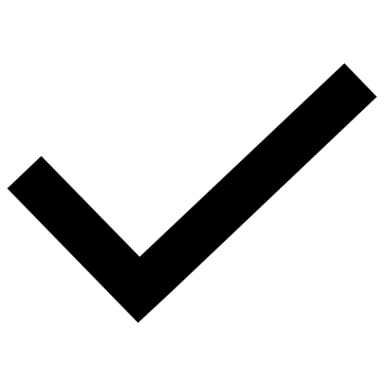 | 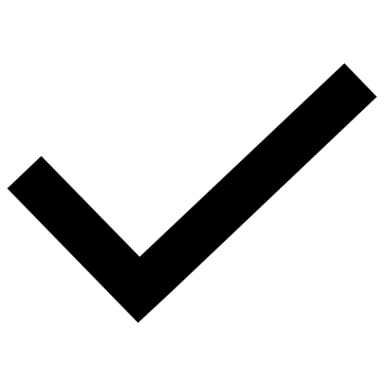 |
|  |  | No | |  |  |  |
|  |  | Unclear | |  |  |  |
|  |  | Not applicable | |  |  |  |
| 8. | Was appropriate statistical analysis used? | Yes | | 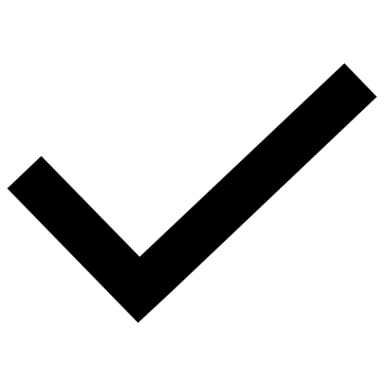 | 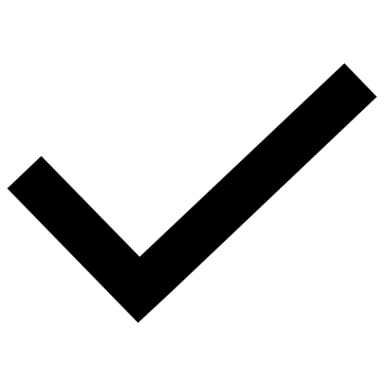 | 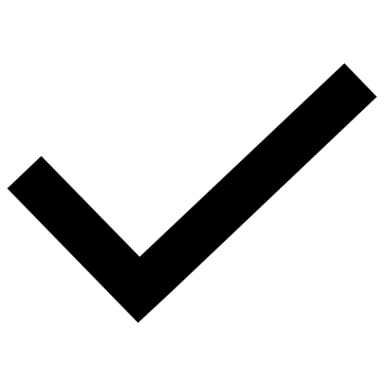 |
|  |  | No | |  |  |  |
|  |  | Unclear | |  |  |  |
|  |  | Not applicable | |  |  |  |


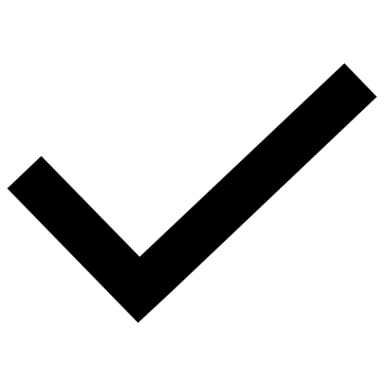
Overall appraisal: Include Exclude □ Seek further info □

Comments (Including reason for exclusion)

**Mixed Methods Appraisal Tool (MMAT), version 2018**

| **Category of study designs** | **Methodological quality criteria** | **Responses** | **Study 1**  Rath 2025 | **Study 2**  Kavadella et al, 2024 |
| --- | --- | --- | --- | --- |
| Screening questions  (for all types) | S1. Are there clear research questions? | Yes | 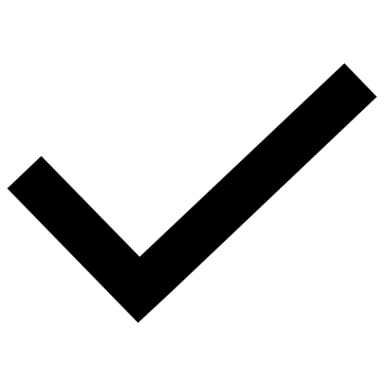 | 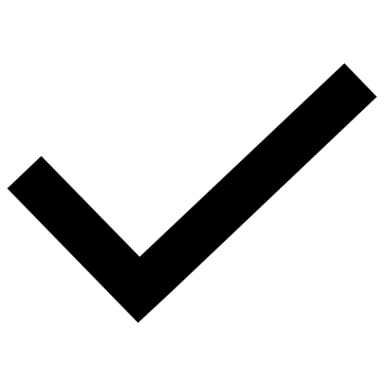 |
|  |  | No |  |  |
|  |  | Can’t tell |  |  |
|  |  | Comments |  |  |
|  | S2. Do the collected data allow to address the research questions? | Yes | 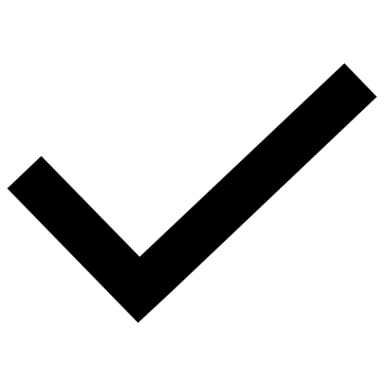 | 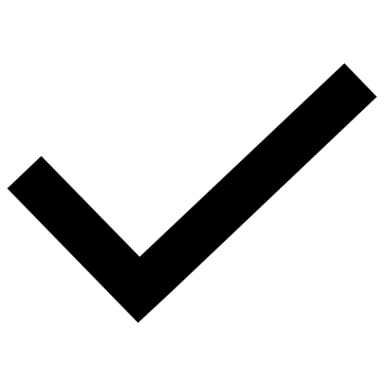 |
|  |  | No |  |  |
|  |  | Can’t tell |  |  |
|  |  | Comments |  |  |
|  | *Further appraisal may not be feasible or appropriate when the answer is ‘No’ or ‘Can’t tell’ to one or both screening questions.* | | | |
| 1. Qualitative | 1.1. Is the qualitative approach appropriate to answer the research question? | Yes | 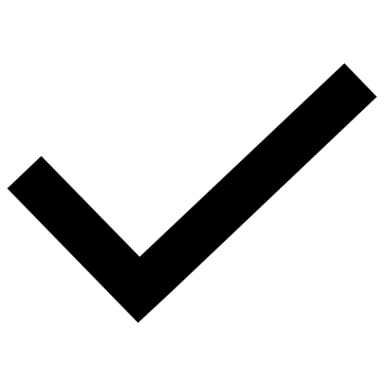 | 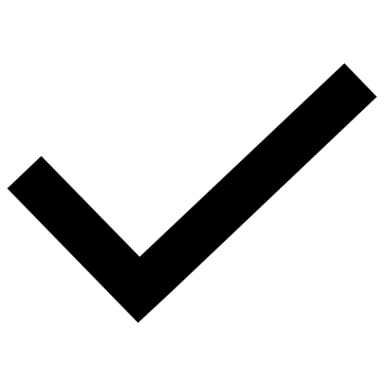 |
|  |  | No |  |  |
|  |  | Can’t tell |  |  |
|  |  | Comments |  |  |
|  | 1.2. Are the qualitative data collection methods adequate to address the research question? | Yes | 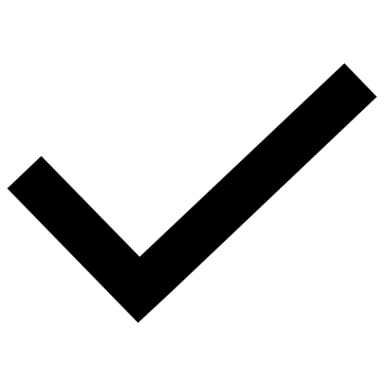 | 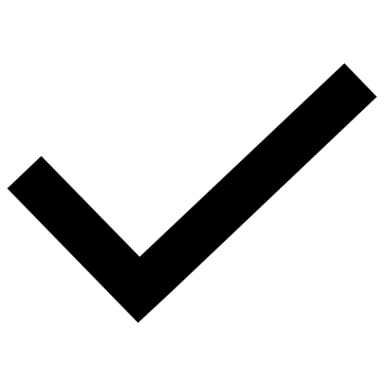 |
|  |  | No |  |  |
|  |  | Can’t tell |  |  |
|  |  | Comments |  |  |
|  | 1.3. Are the findings adequately derived from the data? | Yes | 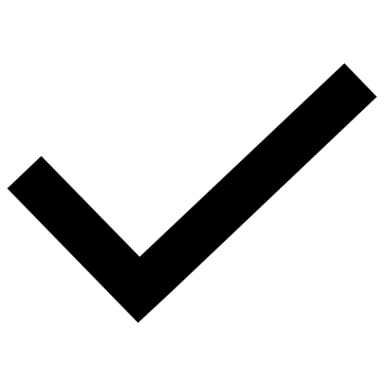 | 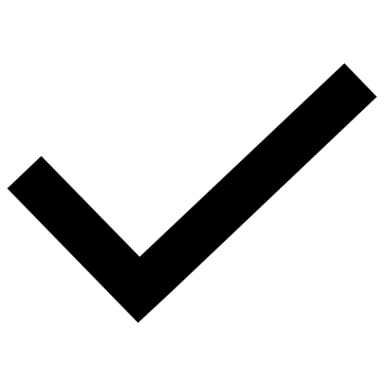 |
|  |  | No |  |  |
|  |  | Can’t tell |  |  |
|  |  | Comments |  |  |
|  | 1.4. Is the interpretation of results sufficiently substantiated by data? | Yes | 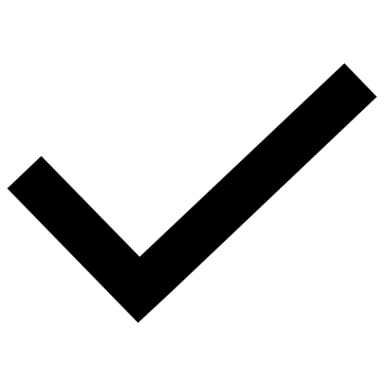 | 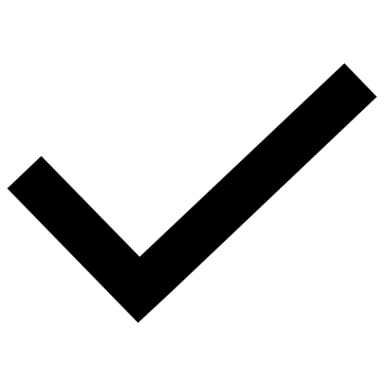 |
|  |  | No |  |  |
|  |  | Can’t tell |  |  |
|  |  | Comments |  |  |
|  | 1.5. Is there coherence between qualitative data sources, collection, analysis and interpretation? | Yes | 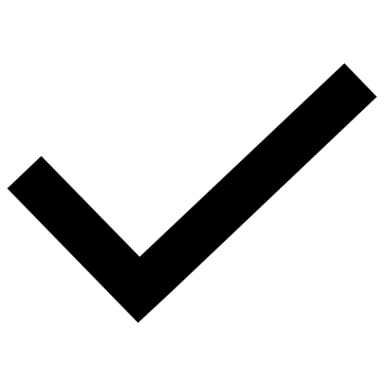 | 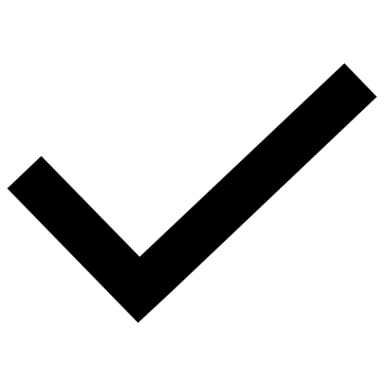 |
|  |  | No |  |  |
|  |  | Can’t tell |  |  |
|  |  | Comments |  |  |
| 2. Quantitative randomized controlled trials | 2.1. Is randomization appropriately performed? | Yes |  |  |
|  |  | No |  |  |
|  |  | Can’t tell |  |  |
|  |  | Comments |  |  |
|  | 2.2. Are the groups comparable at baseline? | Yes |  |  |
|  |  | No |  |  |
|  |  | Can’t tell |  |  |
|  |  | Comments |  |  |
|  | 2.3. Are there complete outcome data? | Yes |  |  |
|  |  | No |  |  |
|  |  | Can’t tell |  |  |
|  |  | Comments |  |  |
|  | 2.4. Are outcome assessors blinded to the intervention provided? | Yes |  |  |
|  |  | No |  |  |
|  |  | Can’t tell |  |  |
|  |  | Comments |  |  |
|  | 2.5 Did the participants adhere to the assigned intervention? | Yes |  |  |
|  |  | No |  |  |
|  |  | Can’t tell |  |  |
|  |  | Comments |  |  |
| 3. Quantitative non-randomized | 3.1. Are the participants representative of the target population? | Yes | 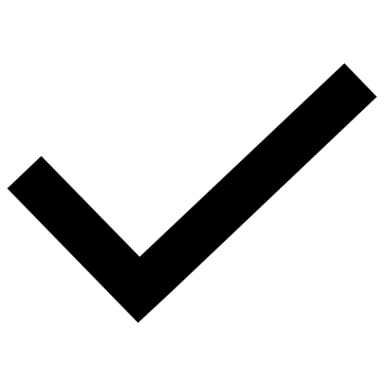 | 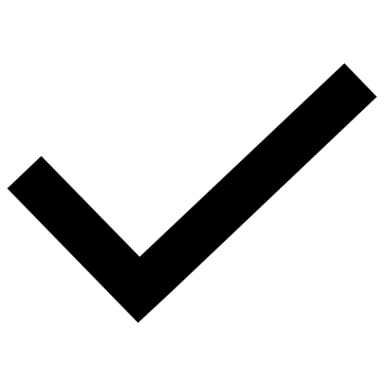 |
|  |  | No |  |  |
|  |  | Can’t tell |  |  |
|  |  | Comments |  |  |
|  | 3.2. Are measurements appropriate regarding both the outcome and intervention (or exposure)? | Yes | 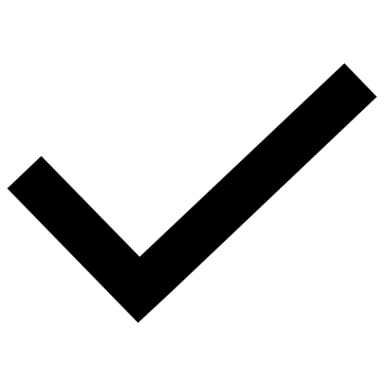 | 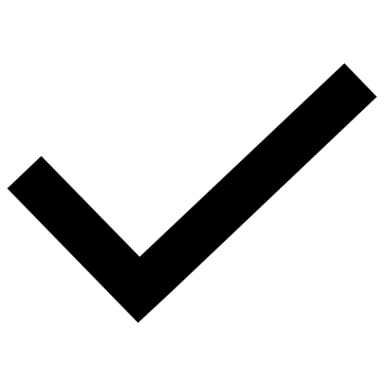 |
|  |  | No |  |  |
|  |  | Can’t tell |  |  |
|  |  | Comments |  |  |
|  | 3.3. Are there complete outcome data? | Yes | 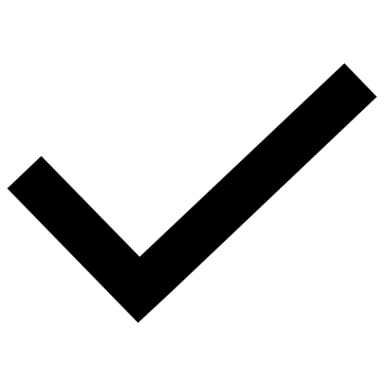 | 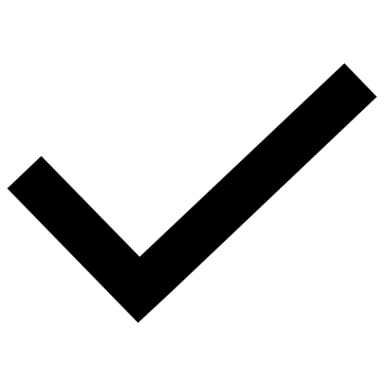 |
|  |  | No |  |  |
|  |  | Can’t tell |  |  |
|  |  | Comments |  |  |
|  | 3.4. Are the confounders accounted for in the design and analysis? | Yes |  |  |
|  |  | No | 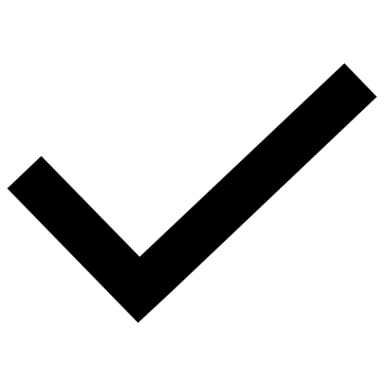 | 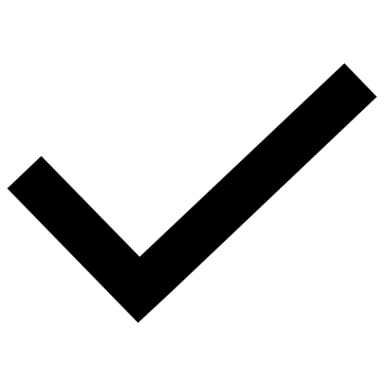 |
|  |  | Can’t tell |  |  |
|  |  | Comments |  |  |
|  | 3.5. During the study period, is the intervention administered (or exposure occurred) as intended? | Yes | 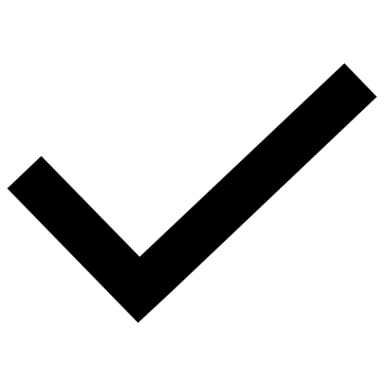 | 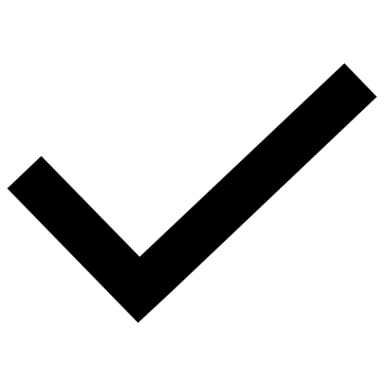 |
|  |  | No |  |  |
|  |  | Can’t tell |  |  |
|  |  | Comments |  |  |
| 4. Quantitative descriptive | 4.1. Is the sampling strategy relevant to address the research question? | Yes |  |  |
|  |  | No |  |  |
|  |  | Can’t tell |  |  |
|  |  | Comments |  |  |
|  | 4.2. Is the sample representative of the target population? | Yes |  |  |
|  |  | No |  |  |
|  |  | Can’t tell |  |  |
|  |  | Comments |  |  |
|  | 4.3. Are the measurements appropriate? | Yes |  |  |
|  |  | No |  |  |
|  |  | Can’t tell |  |  |
|  |  | Comments |  |  |
|  | 4.4. Is the risk of nonresponse bias low? | Yes |  |  |
|  |  | No |  |  |
|  |  | Can’t tell |  |  |
|  |  | Comments |  |  |
|  | 4.5. Is the statistical analysis appropriate to answer the research question? | Yes |  |  |
|  |  | No |  |  |
|  |  | Can’t tell |  |  |
|  |  | Comments |  |  |
| 5. Mixed methods | 5.1. Is there an adequate rationale for using a mixed methods design to address the research question? | Yes | 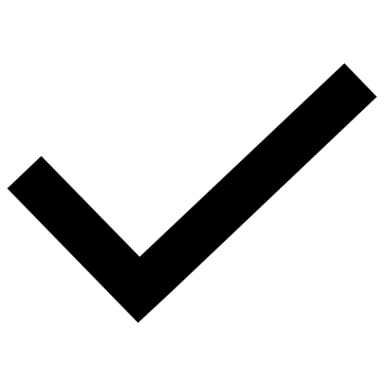 | 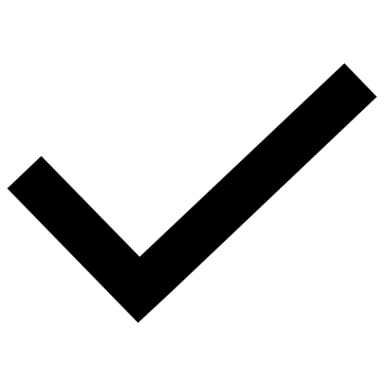 |
|  |  | No |  |  |
|  |  | Can’t tell |  |  |
|  |  | Comments |  |  |
|  | 5.2. Are the different components of the study effectively integrated to answer the research question? | Yes | 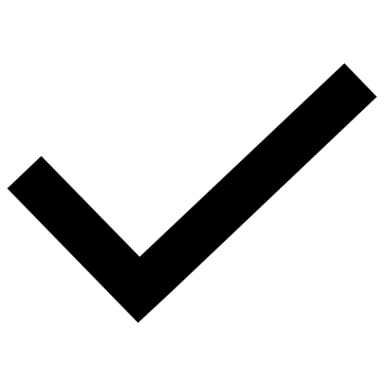 | 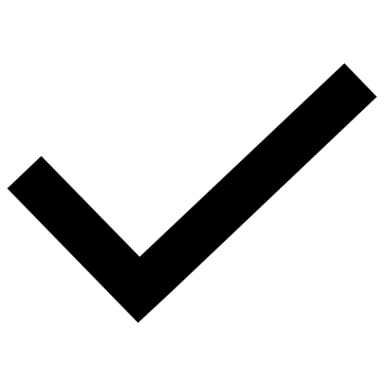 |
|  |  | No |  |  |
|  |  | Can’t tell |  |  |
|  |  | Comments |  |  |
|  | 5.3. Are the outputs of the integration of qualitative and quantitative components adequately interpreted? | Yes | 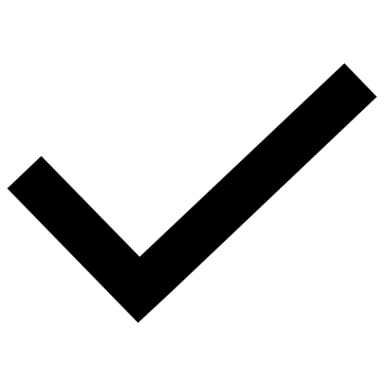 | 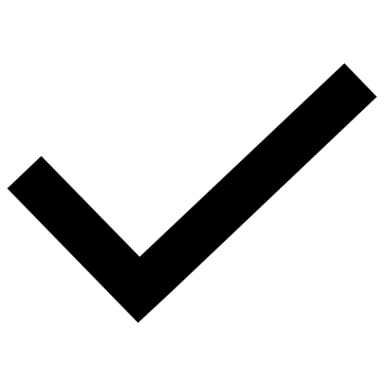 |
|  |  | No |  |  |
|  |  | Can’t tell |  |  |
|  |  | Comments |  |  |
|  | 5.4. Are divergences and inconsistencies between quantitative and qualitative results adequately addressed? | Yes | 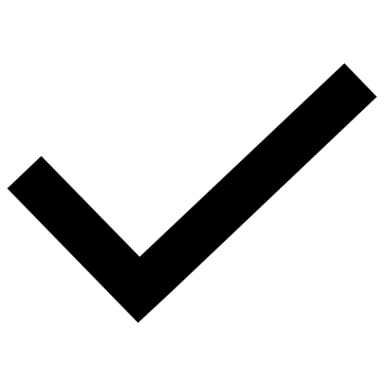 | 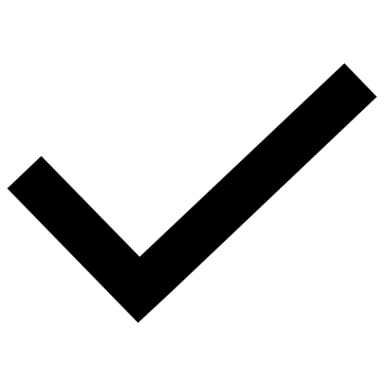 |
|  |  | No |  |  |
|  |  | Can’t tell |  |  |
|  |  | Comments |  |  |
|  | 5.5. Do the different components of the study adhere to the quality criteria of each tradition of the methods involved? | Yes | 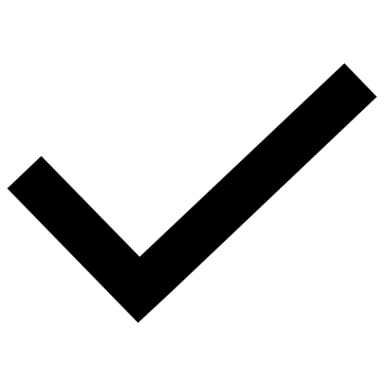 | 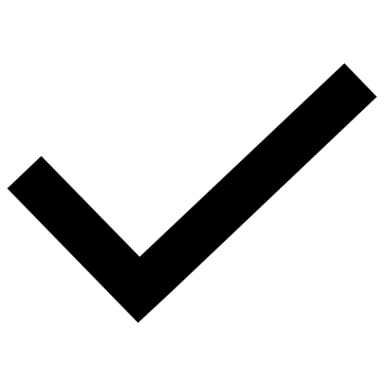 |
|  |  | No |  |  |
|  |  | Can’t tell |  |  |
|  |  | Comments |  |  |

# **SANRA Checklist for Critical Appraisal of Narrative Reviews applied to the narrative review by Dutta A et al, 2024**

The SANRA (Scale for the Assessment of Narrative Review Articles) checklist is a critical appraisal tool designed to assess the quality of narrative review articles. It consists of six items; each scored from 0 (low standard) to 2 (high standard). The total maximum score is 12.

| Sl No. | Checklist Items | Scoring |
| --- | --- | --- |
|  | Justification of the article’s importance for the readership of the journal | 1 |
|  | Statement of concrete aims or formulation of questions | 0 |
|  | Description of the literature search | 0 |
|  | Referencing | 1 |
|  | Scientific reasoning | 1 |
|  | Appropriate presentation of data | 0 |
| Total score | | 3 |

Each item is scored as follows:
0 - Low standard
1 - Medium standard
2 - High standard
